# Single-cell sequencing analysis of multiple myeloma heterogeneity and identification of new theranostic targets

**DOI:** 10.1038/s41419-024-07027-4

**Published:** 2024-09-14

**Authors:** Yanpeng Wang, Yuanliang Peng, Chaoying Yang, Dehui Xiong, Zeyuan Wang, Hongling Peng, Xusheng Wu, Xiaojuan Xiao, Jing Liu

**Affiliations:** 1 Department of Hematology, the Second Xiangya Hospital, School of Life Sciences, Central South University, Changsha, 410011 China; 2 Hunan Province Key Laboratory of Basic and Applied Hematology, Central South University, Changsha, 410011 China; 3 Department of Clinical Laboratory, the Affiliated Nanhua Hospital, University of South China, Hengyang, 421001 China; 4 Shenzhen Health Development Research and Data Management Center, Shenzhen, 518028 China

**Keywords:** Myeloma, Myeloma

## Abstract

Multiple myeloma (MM) is a heterogeneous and incurable tumor characterized by the malignant proliferation of plasma cells. It is necessary to clarify the heterogeneity of MM and identify new theranostic targets. We constructed a single-cell transcriptome profile of 48,293 bone marrow cells from MM patients and health donors (HDs) annotated with 7 continuous B lymphocyte lineages. Through CellChat, we discovered that the communication among B lymphocyte lineages between MM and HDs was disrupted, and unique signaling molecules were observed. Through pseudotime analysis, it was found that the differences between MM and HDs were mainly reflected in plasma cells. These differences are primarily related to various biological processes involving mitochondria. Then, we identified the key subpopulation associated with the malignant proliferation of plasma cells. This group of cells exhibited strong proliferation ability, high CNV scores, high expression of frequently mutated genes, and strong glucose metabolic activity. Furthermore, we demonstrated the therapeutic potential of WNK1 as a target. Our study provides new insights into the development of B cells and the heterogeneity of plasma cells in MM and suggests that WNK1 is a potential therapeutic target for MM.

## Introduction

Multiple myeloma (MM) is a prevalent clinical malignant tumor that originates from the malignant proliferation of plasma cells derived from B lymphocytes and has the second-highest incidence rate among hematological tumors in European and American countries [1]. In the United States, there are approximately 35,000 cases of new diagnosis MM (NDMM) each year, with over 12,000 patients dying from MM [2]. With the emergence of treatment methods such as proteasome inhibitors, immunotherapy, CAR-T cells, autologous hematopoietic stem cell transplantation, and bispecific antibodies, the management of MM has significantly improved [3]. In addition, more than 50% of patients have a 5-year overall survival period [4]. However, MM is still a disease that is difficult to cure at this stage. Therefore, clarifying the pathogenesis of MM and identifying new theranostic targets remain the top priorities in current MM research [5].

Single-cell RNA sequencing (scRNA-seq) technology is a revolutionary technique that allows scientists to better understand the differences between tumors and the microenvironment [6]. Currently, it has been applied in various blood cancers, including leukemia [7, 8], multiple myeloma [9, 10], myelodysplastic syndrome [11], and other highly heterogeneous diseases [12, 13]. Numerous studies have used scRNA-seq to investigate MM. Ling et al. conducted scRNA-seq on bone marrow samples from 9 MM at various Revised International Staging System (R-ISS) stages. They discovered novel molecules (PDIA6, LETM1), networks (UQCRB-PDIA6, STAT1-LETM1), and crosstalk pairs (CCL3/CCL5/CCL3L1-CCR1) [14]. Moreover, Ryu et al. reported abnormal activity in the proliferation, antigen presentation, proteasome, and oxidative phosphorylation of extramedullary myeloma cells in 15 MM, revealing transcriptional programs associated with cell proliferation and immune evasion in invasive MM [15]. Gai et al. combined scRNA-seq data from mouse mononuclear cells with PET-CT and gene expression profile data from 512 NDMM. They discovered that CST6, which is secreted by MM cells, inhibits the development of osteolytic diseases and other diseases that may be related to osteoclast-mediated bone flow in MM by blocking osteoclast differentiation [16]. Liu et al. reported diverse changes in CD4+ T cells in MM response to treatment and recurrence [17]. However, the heterogeneity of tumors has not been fully elucidated, and research on the development of B cells in MM is relatively limited.

Therefore, we used scRNA-seq to analyze B lymphocyte lineage cells, which cover the complete B lymphocyte lineage from hematopoietic stem progenitor cells (HSPCs) to plasma cells, in the bone marrow of MM and healthy donors (HDs). Feature analysis was conducted on 48,293 lineage cells. During the upstream differentiation process of B cells, cytoplasmic translation and RNA splicing were enriched. Mitochondrial-related activity, B-cell activation, and protein folding were enriched in plasma cells. Abnormal communication signals were identified between different cell types in MM through the discovery of new signaling molecules, such as BAFF, PECAM1, VISFATIN, ITGB2, ADGRE5, CADM, and MPZ. The difference in B-cell development between MM and HDs was primarily evident in plasma cells, which is mainly related to various mitochondrial activities. We also identified an important subpopulation of malignant proliferation in MM plasma cells. This subpopulation exhibits gradually increasing proportions during disease progression, high copy number variation (CNV) scores, active cell cycling, high expression of multiple frequently mutated genes, and strong glucose metabolic activity. Furthermore, we identified new theranostic targets, such as WNK1, via kinase omics. The specific inhibitor WNK-IN-11 of WNK1 has shown promising anti-MM effects both in vivo and in vitro. In summary, our work revealed key subpopulations associated with the malignant proliferation of MM plasma cells through scRNA-seq and revealed new theranostic targets.

## Materials and methods

### Bone marrow collection and cell separation

Bone marrow samples from MM patients were taken from Xiangya Second Hospital of Central South University and Hunan Cancer Hospital. Primary human CD34+ cells, CD19+ cells, and CD138+ cells were separated from the bone marrow aspirates of these patients using human CD34, CD19, and CD138 magnetic beads. The collected cells were used to prepare microfluidic droplets. All the samples of human MM patients used in the study were approved as ethical standards by the Ethics Committee of the School of Life Sciences of Central South University and processed in accordance with the approved procedure of the committee.

### Library preparation and sequencing

According to the manufacturer’s instructions, the cell suspension was loaded on a Chromium single-cell controller by using the Single-cell 3’ Library and Gel Bead Kit V2 (10× Genomics, 1000128) and Chromium Single-cell A Chip Kit (10× Genomics, 120236) to generate single-cell gel beads in emulsion (GEMs) [18]. Briefly, individual cells were suspended in 0.04% BSA–PBS. The cells in each channel were loaded, and the target cell recovery rate for each sample was estimated to be 10000 cells. The captured cells were decomposed, and barcodes were used to label the released RNA in a single GEM through reverse transcription (1). GEMs were reverse transcribed in C1000 Touch Thermal Cycles, which were programmed at 53 °C for 45 min, 85 °C for 5 min, and maintained at 4 °C. After reverse transcription, single-cell droplets were destroyed, and single-stranded cDNA was isolated and cleaned with Cleanup Mix (Thermo Fisher Scientific, Shanghai, China) containing DynaBeads. cDNA was generated and amplified, and cDNA quality was evaluated using an Agilent 4200 instrument. Then, according to the instructions of the manufacturer, the scRNA-seq library was prepared using the single-cell 3′ library gel bead kit V2. Finally, sequencing was performed on an Illumina NovaSeq 6000 at a depth of at least 100000 readings per cell, with an opposite end of 150 bp.

### Comparison, quantification, and quality control of scRNA-seq data

The original FASTQ file was mapped to the human reference genome using Cell Ranger 3.0 (10× Genomics). After comparison, a digital gene expression (DGE) matrix was generated for each sample. Merged 10× genome DGE files were generated using the aggregation function of the Cell Ranger pipeline. By balancing the reading depth between libraries, all cells from different batches are merged together for normalization. The final result is the matrix of all cells and their global gene expression.

### scRNA-seq data advanced analysis

Downstream analysis was performed by using the R package Seurat V4.0 [19]. After quality control, normalization, removal of double cells, and other necessary steps, data that do not meet the quality requirements are eliminated. This process results in obtaining standardized data that is suitable for subsequent analysis. Then, the SeuratData dataset and the software packages SingleR [20] and Clusterifyr [21] were used to annotate the clustered data. At the same time, the results of automatic annotation were corrected based on the expression of lineage marker genes. After that, CellChat [22] was used to analyze the cell communication between different cell types, and Monocle2 [23] was used to analyze the differentiation trajectory of B lymphocytes. CytoTRACE2 was used to evaluate this differentiation potential and stemness of the whole cells [24]. Then, the SCENIC workflow was also performed to identify key transcription factors [25, 26]. Finally, the heterogeneity of plasma cell populations was analyzed using InferCNV [27].

### Chemicals and antibodies

WNK-IN-11 (Selleck Chemicals, Shanghai, China) was dissolved in dimethyl sulfoxide (Sigma, Shanghai, China) and stored at −80 °C. Antibodies against GLUT1 (sc-377228, Cruz Biotechnology, USA), IGTB1 (sc-374429, Cruz Biotechnology, USA), WNK1 (28357-1-AP, Proteintech, China), PECAM1(sc-20071, Cruz Biotechnology, USA), CD74 (sc-6262, Cruz Biotechnology, USA), and GAPDH (sc-47724, Santa Cruz Biotechnology, Shanghai, China) were used.

### Cell and cell culture

ARP1 and MM.1S cell lines were obtained from the Institute of Hematology and Blood Diseases Hospital, Chinese Academy of Medical Science & Peking Union Medical College, Tianjin, China. ARP1 and MM1S cells were identified by short tandem repeat profiling and cultured in RPMI 1640 (Gibco, Shanghai, China) supplemented with 10% fetal bovine serum (Sigma, Shanghai, China), 2 mM glutamine, 100 U/mL penicillin, and 100 μg/mL streptomycin at 37 °C in the presence of 5% CO_2_ in a humidified chamber.

### Cell viability, proliferation, and cell cycle

According to the manufacturer’s protocol, cell viability was determined using a Cell Counting Kit (CCK)-8. The absorbance was measured at 450 nM using a Randox Toxicology microplate reader. Cell proliferation was manually assessed using a trypan blue exclusion assay and soft agar clonogenic assay. After staining with the Cell Cycle Staining Kit, a FACSCalibur (BD Biosciences, Shanghai, China) flow cytometer was used to assess the cell cycle distribution.

### Quantitative real-time polymerase chain reaction

Total RNA was extracted with TRIzol (Vazyme, Nanjing, China,) using the standard procedure. RNA was then reverse transcribed into cDNA using the HiScript® Q RT SuperMix for qPCR Kit (Vazyme, Nanjing, China). qRT-PCR was performed using a Mastercycler® ep realplex system (Eppendorf, Germany), and mRNA expression levels were then calculated using the 2^−^^ΔΔCT^ method. The primers used are for WNK1: forward 5′-CTGGAACTGCTCCCTCCAAG-3′, reverse 5′-CACCCTTCTGAGGCTGTGTT-3′, GAPDH: forward 5′- TTTGCGTCGCCAGCCG-3′, reverse 5′-ACGGTGCCATGGAATTTGCC-3′.

### Lentivirus infection

WNK1-specific short hairpin RNA (shRNA) virus was purchased from GenePharma (Shanghai, China) (shRNA #1: 5′-GCCAGTACCAACTATCCAAGG-3′ and #2: 5′-GCAACAAGCAGCCCTCCTAAT-3′). A total of 3 × 10^6^ lentiviral particles were mixed with polybrene and added to 5 × 10^5^ cells for transduction. Puromycin (1 μg/ml) was used for the selection of transduced cells.

### Cell membrane isolation and Western blotting

For the isolation of the cell membrane, MM cells were harvested and suspended in a hypotonic lysing buffer containing 20 mM Tris-HCl (pH 7.5), 10 mM KCl, 2 mM MgCl_2_, and 1 mM EDTA-free protease inhibitor per 10 mL of solution. Then the entire cell suspension was disrupted with 20 passes using a Dounce homogenizer, and the supernatant was collected after centrifugation at 3200*g* for 5 min. The pellet at the bottom was resuspended in the hypotonic lysing buffer to repeat the aforementioned steps. The supernatants were pooled and centrifuged at 20,000*g* for 20 min. Subsequently, the supernatant was centrifuged again at 100,000*g* for 1.5 h, and the resulting pellet was resuspended in RIPA buffer (Thermo Fisher Scientific, Shanghai, China) supplemented with protease inhibitors and PhosStop (Roche, Switzerland). The protein concentration was quantified using the Pierce Bicinchoninic Acid Protein Assay Kit (Thermo Fisher Scientific, Shanghai, China). Equal amounts of proteins were loaded into each lane of the gel, and then transferred to a membrane. The membrane was incubated with primary antibodies, and then the appropriate secondary antibodies conjugated to HRP were added. A SuperSignal West Pico substrate (Thermo Fisher Scientific, Shanghai, China) chemiluminescence kit and a Gene Genius bioimaging system were used to detect the protein bands.

### MM xenograft mouse model

All experiments in vivo were approved by the Ethics Committee of the School of Life Sciences of Central South University. And investigators were blinded to the randomization. Female NCG mice, 4–6 weeks, were purchased from GemPharmatech (Nanjing, China). For Fig. 6A–G, a total of 5 × 10^6^ ARP1-LUC cells were inoculated subcutaneously into the backs of the mice. When the tumors could be measured, the mice were randomly divided into three groups and given an equal volume of PEG300 + Tween 80 (control group), WNK-IN-low (1 mg/kg treatment group), or WNK-IN-high (5 mg/kg treatment group). The injections were administered intraperitoneally every two days for a total of eight days. Tumor volume and body weight were measured daily. Mice were imaged on the last day using a Carestream In-Vivo MS FX Pro. The mice were euthanized at the end of the experiment. The tumors were quickly removed, and the weight and grouping of the tumors were recorded. Photos were taken after being arranged. Tumors were fixed in 10% formaldehyde and embedded in paraffin. Immunofluorescence staining was used for detection. A Nikon fluorescence microscope was used for image acquisition. For Fig. 6H–J, the NCG mice were injected with 1 million ARP1-LUC cells via the tail vein to enable full-body dissemination. Seven days after the injection, the mice were randomly divided into two groups. One group received an equal volume of PEG300+Tween 80 (control group), while the other group received WNK-IN-11 (5 mg/kg treatment group). The injections were administered intraperitoneally every two days. After 20 days, the mice were imaged using a Carestream In-Vivo MS FX Pro. The exposure time was kept constant in all images, and the tumor burden was analyzed by selecting areas around the mice and quantifying the pixel intensity for each mouse in each group. The mice were kept alive for survival analysis.

### Statistical analysis

The Wilcoxon signed rank test and *t* test were used for paired comparisons. Prism 8 was used to present the data in bar graphs, with all values presented as the means ± standard deviations (SD). Comparisons between the two groups were performed using an unpaired, two-tailed *t* test. Multiple group comparisons were performed using one-way ANOVA. *p < 0.05, **p < 0.01, and ***p < 0.001.

## Results

### Construction of the B lymphocyte lineage transcriptome map via scRNA-seq

**Fig. 1:**
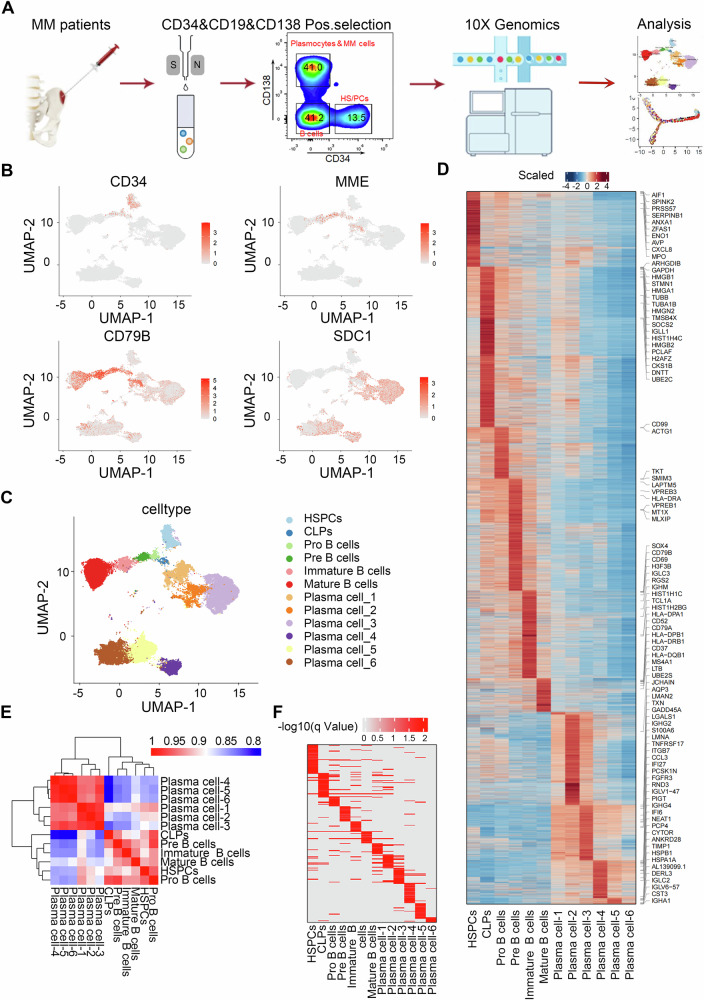
Construction of the B lymphocyte lineage expression profile of MM patients. **A** Workflow. Bone marrow samples from 7 patients with multiple myeloma were selected for scRNA-seq. CD34+/CD19+/CD138+ cells enriched by MACS were further sorted and detected using flow cytometry. Cells were analyzed using the 10× Genomics Chromium scRNA-seq platform. **B** Expression of four characteristic marker genes (CD34, MME, CD79B, and SDC1) during B-cell development. **C** Unified manifold approximation and projection (UMAP) representation of single-cell gene expression, showing the 12 identified cell types. Cells were color-coded based solely on cluster annotation. **D** Expression heatmap of characteristic marker molecules for each cell subset. **E** Correlation analysis map of each cell subset. **F** BP enrichment results of characteristic expressed genes for each cell subset.

MM is a clonal disease characterized by the malignant proliferation of plasma cells at the final stage of B-cell development [5]. To better understand the pathogenesis of MM, fresh bone marrow samples were obtained from 7 MM (Table S1). CD34, CD19, and CD138 magnetic beads were used to sort B lymphocyte lineage cells and performed scRNA-seq with 10× Genomics (Fig. 1A).

To analyze the differences between MM and HDs, we utilized bone marrow samples from eight normal individuals in the public Human Cell Atlas (HCA) database as normal controls [28]. After removing low-quality cells (Fig. S1), unsupervised clustering was performed on 49,867 B lymphocyte lineage cells from 7 MM and 8 HDs, creating a map consisting of 29 cell clusters (Fig. S2A). Although the expression of biomarkers such as CD34, MME, CD79B, and SDC1 indicated a relatively high purity of enriched cells (Fig. 1B), analysis of the transcriptome expression characteristics of each cell cluster revealed that multiple clusters still showed contamination from non-B lymphocyte lineage cells (Fig. S2B and Table S2).

After removing the contaminated cell clusters, we ultimately obtained 7 continuous B lymphocyte lineage cell clusters (Fig. 1C and Fig. S2C): HSPCs, common lymphoid progenitors (CLPs), Pro B cells, Pre B cells, immature B cells, mature B cells, and plasma cells. Due to the strong heterogeneity of plasma cells, the cells were divided into 6 subpopulations. Each cell type has its own unique expression profiles of characteristic genes such as *CXCL8*, *SOCS2*, *LAPTM5*, *CD37*, and other characteristic genes that play important roles in the development of B cells (Fig. 1D and Table S3).

By comparing the gene expression of each subgroup and analyzing the correlation among the cell types, it was found that plasma cells exhibited significant differences from the other six cell types (Fig. 1E). In addition, those plasma cell clusters were roughly divided into two heterogeneous groups, and there was a certain similarity in the distribution of the six subpopulations according to the UMAP results (Fig. 1C). By combining the characteristic genes of each subgroup, we found through Gene Ontology (GO) enrichment analysis that during the upstream differentiation process of B cells, cytoplasmic translation and RNA splicing were enriched. Mitochondrial-related activity, B-cell activation, and protein folding were enriched in plasma cells (Fig. 1F, Fig. S3, and Table S4).

### The alteration of cellular communication in MM

**Fig. 2:**
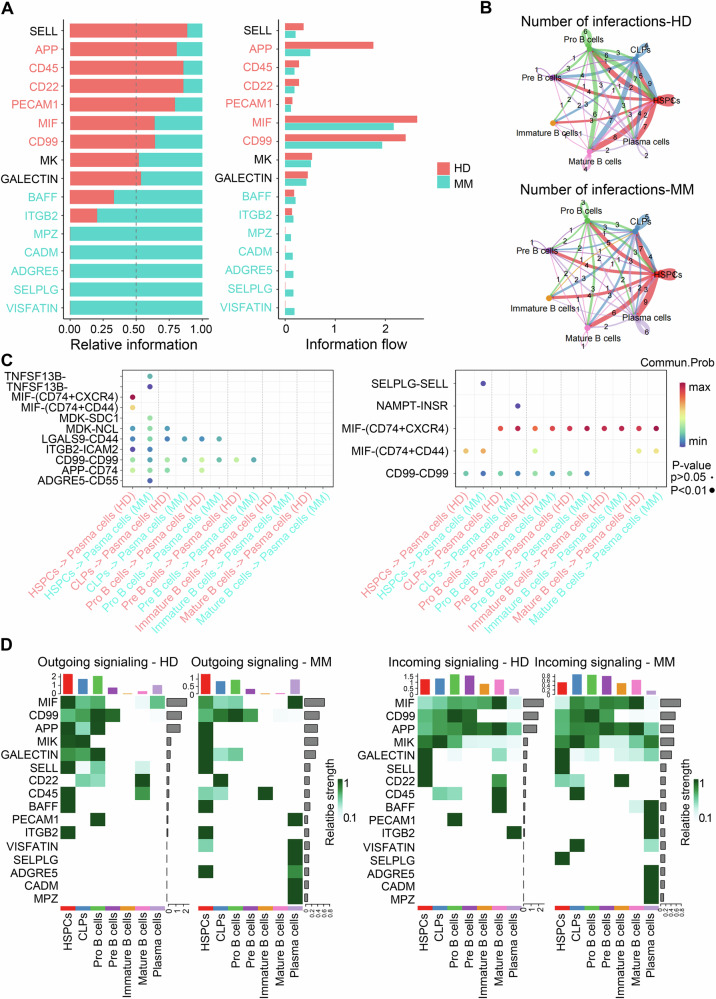
Cellular communication between cell clusters. **A** The communication signals differed between HDs and MM patients. The left panel shows the proportions of various signaling molecules in HDs and MM patients, while the right panel shows the levels of different signaling molecules detected in HDs and MM patients. **B** Differences in signal molecule pairs between HDs and MM patients. **C** Differences in molecule pairs involved in plasma cell input and output signaling between HDs and MM patients. **D** Differences in the input and output signals between the HDs and MM patients. On the left side, the figure displays the expression of output signaling molecules in each cell cluster of HDs versus MM patients, while the right side illustrates the expression of input signaling molecules in each cell cluster of HD patients versus MM patients.

CellChat was used to analyze the connections among the cell types and investigate the potential differences in cell communication between MM and HDs [29]. It was found that the intercellular communication of MM changed significantly (Fig. 2A). And the expression levels of signaling molecules such as SELL, APP, CD45, CD22, PECAM1, CD99, and MIF were significantly reduced in MM. Moreover, MM patients exhibited unique signaling molecules, such as VISFATIN, SELPLG, ADGRE5, CADM, and MPZ, which were almost exclusively detected in MM. We calculated the ligand‒receptor interaction strength between different lineages of B lymphocytes (Fig. 2B, C). The number of signaling pairs increased from 2 pairs to 6 pairs in the plasma cells of MM patients. In addition, HSPCs were closely associated with other celltypes in both MM and HDs, However, further research is needed to determine the specific role of HSPCs in cell communication. We found that CD74–CXCR4 and CD74–CD44 were involved in communication between plasma cells (Fig. 2C). This finding is consistent with previously reported studies on CD74 in MM [30, 31]. We also discovered that the majority of the identified signaling molecules (PECM1, NAMPT, SELPLG, ADGRE5, CADM, and MPZ) were associated with plasma cells by analyzing the input and output signals of each cell cluster (Fig. 2D). These signaling molecules may have an important impact on the malignant proliferation of plasma cells.

### The developmental trajectory of the B lymphocyte lineage in MM

**Fig. 3:**
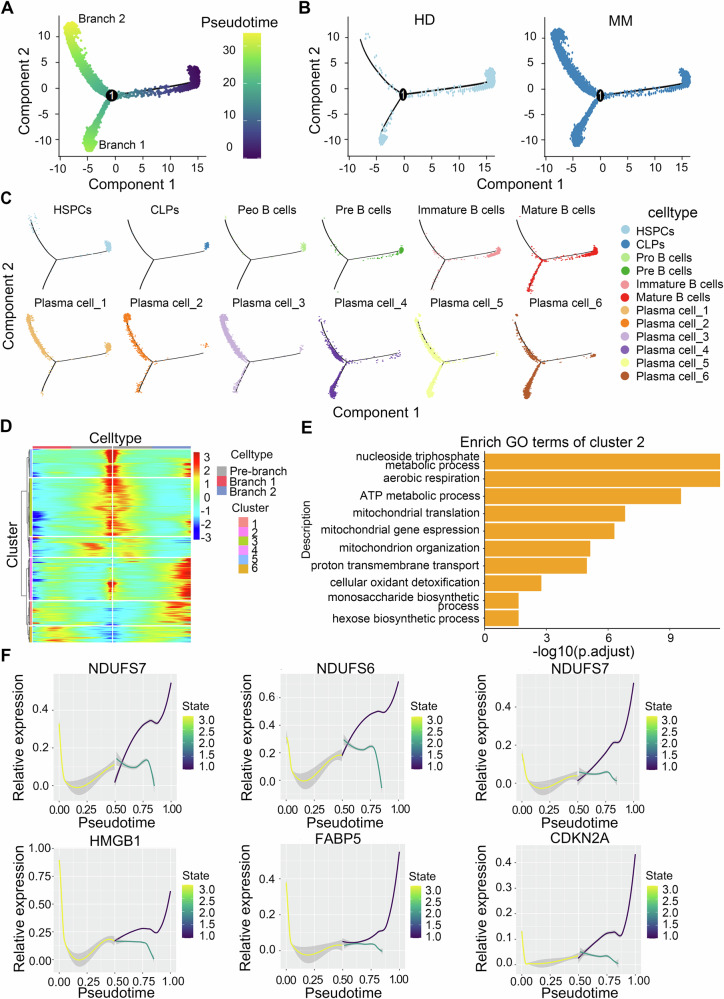
The developmental trajectory of B cells in MM. **A** Pseudotemporal analysis scores of expression profiles. **B** Pseudotemporal analysis of divergence trajectories between HDs and MM patients. **C** Pseudotemporal analysis of the divergent trajectories of each subgroup. **D** Heatmap showing gene enrichment on the two branches after the bifurcation point. **E** Results of Gene Ontology Biological Process (GO-BP) analysis of differences in Cluster 2. **F** Expression of the mitochondria-related genes NDUSF2, NDUSF5, and NDUDF7; the cycle-related gene CDKN2A; the autophagy-related gene HMGB1; and the lipid metabolism-related gene FABP5 according to the differentiation trajectory of the expression profile.

Studies have reported that disturbances in intercellular communication can lead to abnormalities in the development process [32]. Based on the huge differences in cell communication between various cell types between MM and HD before. Then Monocles2 was used to track the developmental trajectories of HSPCs, CLPs, Pro B cells, Pre B cells, immature B cells, mature B cells, and plasma cells in seven consecutive stages (Fig. 3A–C). As expected, HSPCs and B cells are orderly arranged in pseudotime sequence, and plasma cells are divided into two distinct branches. We compared the differentiation trajectories of HDs and MMs, and the main difference was in branch 2 (Fig. 3B). The main difference between HDs and MMs was the distribution of plasma cells. According to the distribution of plasma cell subpopulations, the cells in branch 2 were mainly composed of plasma cell_2 and plasma cell_3 (Fig. 3C). By clustering the all genes on the trajectory of pseudotime analysis, we found that the gene expression of Cluster 2 was significantly upregulated in branch 2 (Fig. 3D). We conducted GO enrichment analysis on the gene set of Cluster 2 and found that the enriched pathways were mainly related to mitochondrial functions (Fig. 3E and Table S5). We determined the expression of multiple mitochondrial-related genes, such as NDUFS7, NDUFS6, and NDUFS2, along the developmental trajectory and observed significant differences in these genes after passing through branching points (Fig. 3F). In addition, many genes, such as CDKN2A, HMGB1, and FABP5, which are significantly negatively associated with the prognosis of MM, exhibited notable differences in expression between the two branches.

### The heterogeneity of plasma cells in MM

**Fig. 4:**
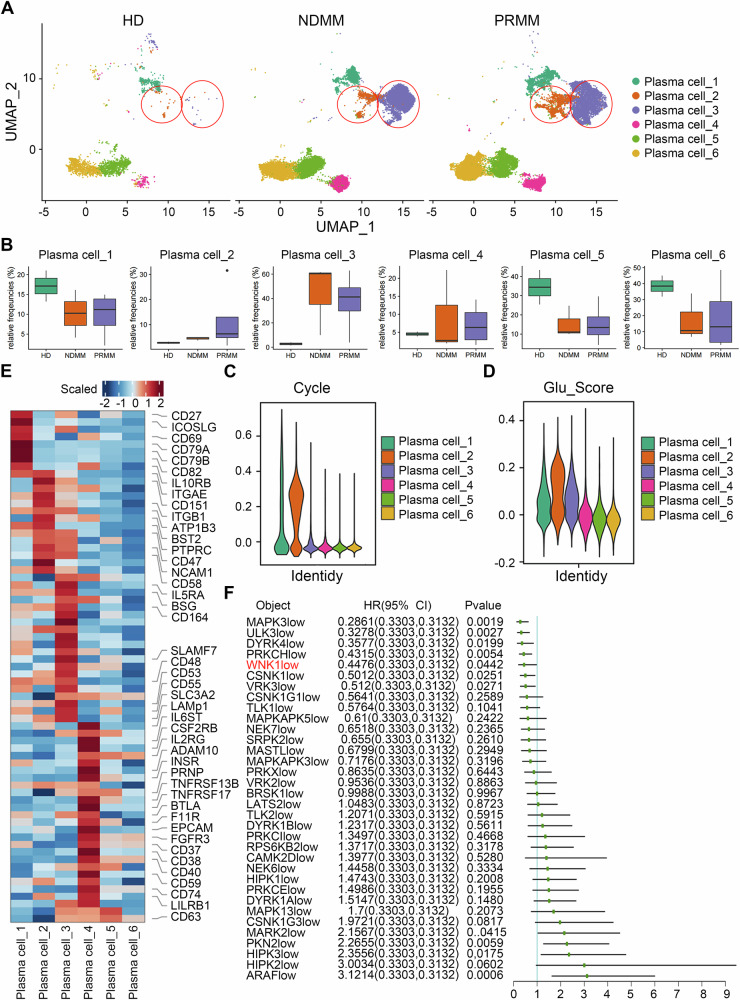
Heterogeneity analysis of plasma cells. **A** UMAP plot illustrating the distribution of plasma cell subsets in HDs, NDMMs, and RPMMs. **B** The proportion of each plasma cell subset in HDs, NDMMs, and RPMMs. **C** The heterogeneity of cell cycle proliferation and division ability in each plasma cell subset. **D** Heterogeneity of glucose metabolism capacity in plasma cell subsets. **E** Expression of characteristic CD molecules in plasma cell subsets. **F** Survival curves of MM patients and correlation analysis of kinases and MM patients prognosis.

Based on the previous results, the main difference between MM and HD is primarily seen in plasma cells. Therefore, we carried out a further analysis of the heterogeneity of plasma cells. The plasma cells were classified into 6 subpopulations based on their characteristic gene expression during cell clustering (Fig. 4A). The percentage of plasma cell_2 and plasma cell_3 were increased in MM compared with HDs (Fig. 4B). Furthermore, they were almost only detected in the MM, and its proportion of plasma cell_2 was significantly increased in progression relapsed MM (PRMM) compared with NDMM.

Cytogenetic analysis revealed multiple gene mutations in MM, with the majority concentrated in structural rearrangements and copy number abnormalities [33]. Currently, common CNVs include a gain of 1q, deletion of 1p, deletion of 13q, and deletion of 17p [34]. We analyzed the CNV status of HD and MM plasma cells using inferCNV. Consistent with previous reports, the CNV score of MM plasma cells was significantly higher than that of HD plasma cells (Fig. S4A, B). Among the six plasma cell subsets, plasma cell group 2 had the highest CNV score (Fig. S4C). We detected significant CNVs on chromosomes 1, 5, 6, 7, 9, 14, 15, 17, 19, 20, and 22 (Fig. S4D). The gain on chromosome 1 and the deletion on chromosome 17 are consistent with those previously reported.

We also evaluated the differentiation potential of plasma cell subpopulations using CytoTRACE2. The results showed that the differentiation potential of plasma cell_1, plasma cell_2, and plasma cell_3 was significantly higher than that of the other 3 subpopulations (Fig. S5). Furthermore, we used the SCENIC package to detect transcription factors that are highly expressed in subpopulations of plasma cells. It was found that the transcription factors TLX2, FOXJ1, SOX11, HMGA1, and ZNF560 were significantly overexpressed in plasma cell_2 (Fig. S6). Among them, HMGA1 plays a crucial role in the extramedullary progression of MM [35].

We determined the expression of 254 highly frequently mutated genes in various subpopulations of plasma cells. The results showed the expression of tumor malignant characteristic genes such as *CDKN2A CKS1B*, *HSPA9*, *IDH2*, and *MYC* among different subpopulations of plasma cells [36–40], with high expression in plasma cell_2 (Fig. S7). Importantly, we detected heterogeneity in multiple components of proteasomes and PABPC1, which is involved in alternative polyadenylation, among the different subpopulations of plasma cells. This is consistent with what has been reported [41, 42].

Malignant proliferation and active glucose metabolism are also important characteristics of MM cells [43]. We analyzed the proliferation ability of each plasma cell subpopulation by cell cycle scoring function and module analysis. The results showed that plasma cell_2 exhibited the highest proliferation ability (Fig. 4C and Fig. S8), which is consistent with previous results (Fig. S5). In addition, we determined the expression of key genes involved in glucose metabolism in each subpopulation and calculated the glucose metabolism score in each subpopulation through modular analysis. The results showed that the glucose metabolism capacity of plasma cell_2 was the highest (Fig. 4D and Fig. S9). Therefore, plasma cell_2 might be the most malignant subpopulation.

We analyzed the expression of membrane surface protein in different subpopulations of plasma cells to discover novel tumor markers or potential diagnostic and therapeutic targets related to MM. BST2, CD6, CD58, PTPRC, NCAM1, and CD56 were significantly overexpressed in plasma cell_2 (Fig. 4E, Table S6). We previously defined plasma cell_2 as a highly malignant heterogeneous population. These membrane surface molecules, which are significantly overexpressed in this group, can be considered diagnostic markers for detecting this malignant group in clinical practice.

We also analyzed the expression profiles of kinases in various plasma cell subpopulations and found that each subpopulation had a large number of specifically expressed kinases, such as SGK1, CDK2, CDC7, and CDK5 (Fig. S10, Table S6). Most kinases belong to the serine/threonine kinase family. By searching for new therapeutic targets combined with clinical prognostic databases, we found that 13 kinases were negatively correlated with MM prognosis (Fig. 4F). These 13 kinases are potential therapeutic targets for MM. These kinases are WNK1, MAPKAPK5, MARK2, MARK3, MASTL, NEK7, PRKCH, PRKCI, PRKX, SRPK2, TLK2, VRK2, and VRK3.

### The biological function of WNK1 in MM cells

**Fig. 5:**
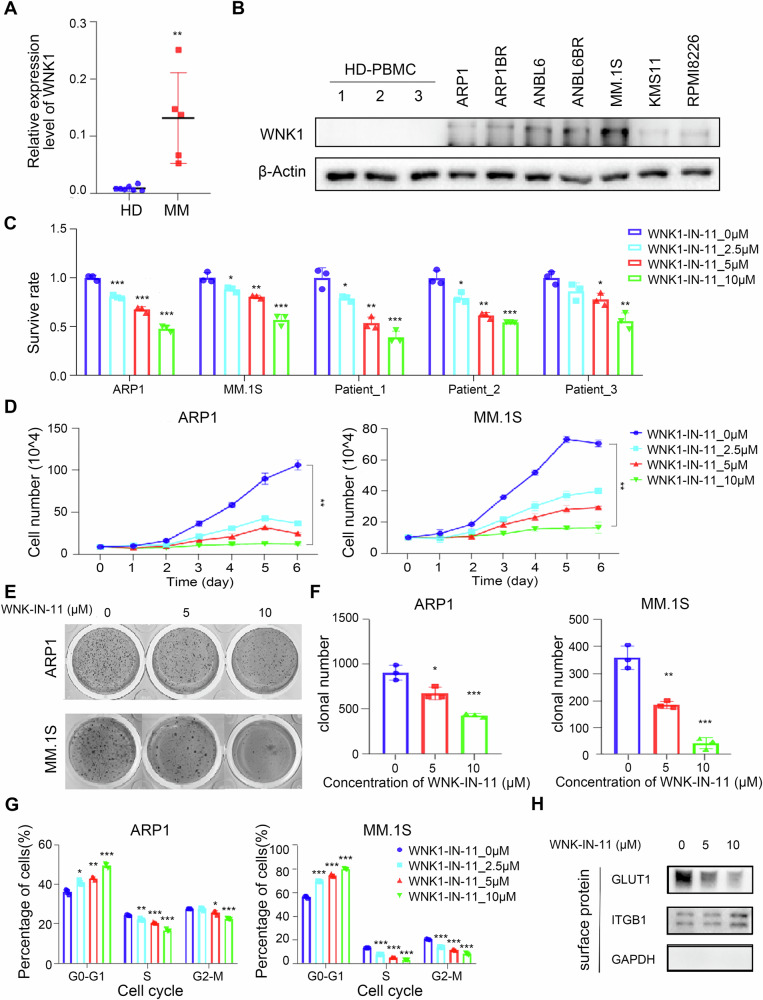
Biological effects of WNK1 on MM cells. **A** Expression levels of WNK1 in the remaining bone marrow samples from normal donors and MM patients were determined using qRT-PCR. **B** The expression levels of WNK1 in the remaining bone marrow samples from normal donors and MM cell lines were detected using Western blot analysis. **C** Cell viability analysis of MM cell lines and bone marrow samples from MM patients. **D** Growth curves of MM cell lines treated with WNK-IN-11. **E** Colony formation experiments were conducted on MM cell lines treated with WNK-IN-11. **F** Statistics of clone numbers of MM cell lines. **G** Effect of WNK-IN-11 on the cell cycle distribution of MM cell lines, as determined by flow cytometry. **H** GLUT1 expression on the membrane surface was determined. GAPDH was used as the internal reference for plasma proteins, and ITGB1 was used as the internal reference for membrane proteins. Statistical analyses of *n* = 3 independent experiments were assessed. *p < 0.05, **p < 0.01, ***p < 0.001.

As shown above results, the kinases may play an important role in the occurrence and development of MM. Consequently, we detect the expression of those kinases in the collected clinical samples. We found that *WNK1* is highly expressed in MM patient’s mRNA levels, and WNK1 is also expressed in various MM cell lines’ protein levels (Fig. 5A, B). These findings further indicate that WNK1 may play an important role in MM. We treated the MM cell lines ARP1 and MM.1S with shRNA-WNK1 or WNK-IN-11, a specific inhibitor of WNK1 [44]. We observed that the activity and proliferation of MM cell lines were inhibited (Fig. 5C–F and Fig. S11). Flow cytometry analysis revealed that inhibiting WNK1 activity can result in cell cycle arrest, by blocking the MM cell cycle in the *G*_0_/*G*_1_ phase (Fig. 5G). Furthermore, WNK-IN-11 can significantly decrease the localization of GLUT1 on the MM cell membrane (Fig. 5H and Fig. S12). WNK-IN-11 can also reduce the protein expression level of cell communication molecules PECAM1 and CD74 by Western Blot assay (Fig. S13). In addition, the activity of bone marrow mononuclear cells from MM patients was significantly inhibited after WNK-IN-11 treatment (Fig. 5C).

### WNK-IN-11 plays an anti-MM role in vivo

**Fig. 6:**
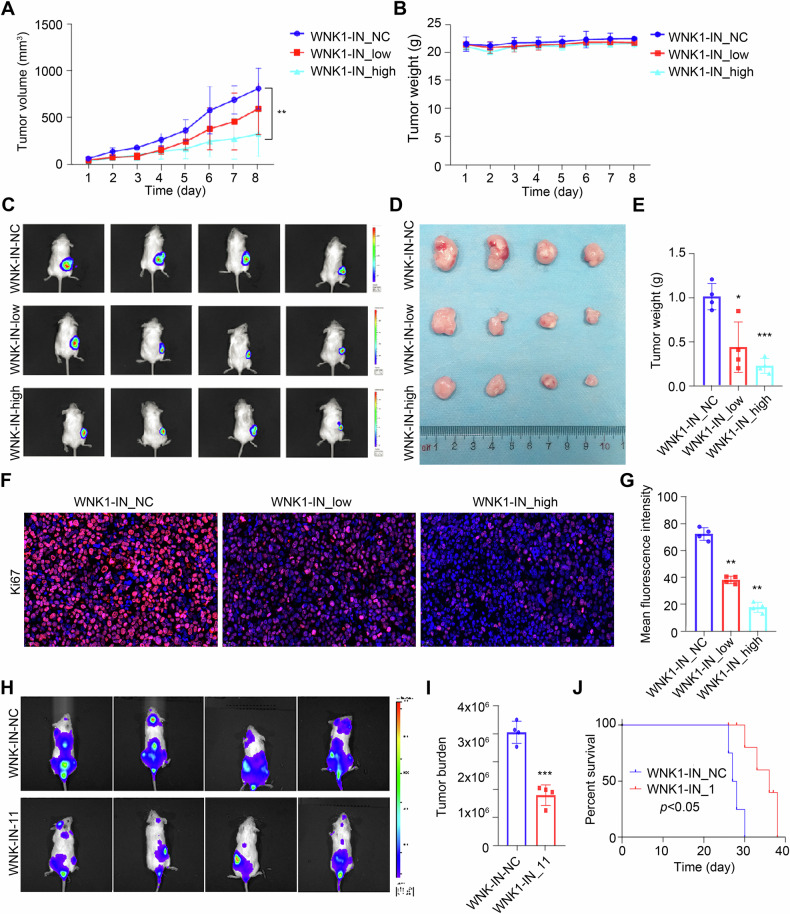
WNK-IN-11 has anti-MM effects in vivo. **A** Tumor volume change curve. **B** The body weights of the mice remained stable. **C** Fluorescence imaging results of subcutaneous tumors. **D** Tumor growth differences between groups. **E** Statistical analysis of the tumor weight data. **F** Ki67 immunohistochemical staining of tumors from each group was performed. **G** Statistical analysis of fluorescence intensity data. **H** Fluorescence imaging showing tumor development after tail vein injection. **I** Statistical analysis of the fluorescence intensity. **J** Survival curve of the mice. WNK-IN-low is a low-dose group with a concentration of 1 mg/kg. WNK-IN-high is a low-dose group with a concentration of 5 mg/kg. *p < 0.05, **p < 0.01, ***p < 0.001.

Furthermore, we explored the therapeutic effect of WNK-IN-11 on MM in vivo, by using an NCG mouse subcutaneous tumorigenesis model with fluorescein-labeled ARP1-LUC cells [45]. When a tumor mass was observed at the site of tumor formation, WNK-IN-11 (1 mg/kg and 5 mg/kg) was injected intraperitoneally every two days, and the weight and tumor volume of mice were weighed every day. As expectedly, WNK-IN-11attenuated significantly the rate of tumor growth and volume (Fig. 6A). And WNK-IN-11 also has no remarkable effect on the weight of tumor-bearing mice (Fig. 6B). Fluorescence imaging of mice and observation of stripped tumors showed that WNK-IN-11 treatment significantly inhibited tumor growth (Fig. 6C–E). Immunohistochemical staining results of the tumors showed that the expression of Ki67 in the tumor tissue decreased with increasing WNK-IN-11 dose (Fig. 6F, G). These results all indicated that WNK-IN-11 had a significant therapeutic effect on the subcutaneous tumorigenesis mouse model.

In addition, we constructed a systemic tumor formation model of NCG using tail vein injection of ARP1-LUC cells and intraperitoneal injection of WNK-IN-11. After 20 days of treatment, the WNK-IN-11 treatment significantly inhibited tumor progression, reduced tumor burden, and prolonged the survival of tumor-bearing mice compared to those in the control group (Fig. 6H–J). These findings fully demonstrated that WNK-IN-11 has an inhibitory effect on MM in vivo.

## Discussion

scRNA-seq technology can be used to analyze the intercellular variations between tumors and the microenvironment, providing a deeper understanding of the pathogenic mechanisms of diseases. At present, scRNA-seq technology has been applied in multiple studies, but the heterogeneity of MM has not yet been thoroughly studied. Moreover, it is still unclear whether plasma cell abnormalities in MM can cause the abnormal development of B cells. In our study, we performed scRNA-seq using HSPCs, B cells, and plasma cells from MM patients. Multiple subpopulations of B lymphocyte lineages were identified. We described the basic characteristics of the cell subpopulations and their interrelationships and further analyzed the heterogeneity of the plasma cells. This study aimed to identify new theranostic targets through in vitro and in vivo experiments.

Through CellChat, we found that the communication between B lymphocyte lineages of MM patients was disrupted. Furthermore, a variety of signaling molecules were found to be significantly upregulated. Previous studies have shown that these signaling molecules can regulate the biological processes of target cells [46]. Therefore, the signaling molecules upregulated in MM can be used as potential therapeutic targets.

We utilized pseudotime analysis to map the developmental trajectory of the B lymphocyte lineage from HSPCs to plasma cells for the first time and investigate the disparities in B-cell development between MM and HDs. Only a few of these stages have been involved in the reported studies [17, 47]. Our results showed that the main differences were reflected in the plasma cells. Through functional enrichment analysis, it was found that the differences were primarily in the production of mitochondrial energy. Currently, multiple studies have attempted to address the challenge of treating MM by targeting mitochondria. For example, the use of electron transport chain inhibitors to treat MM resulted in the upregulation of the cysteine glutamate reverse transporter SLC7A11 in MM, thereby reducing mitochondrial stress-induced proteasome inhibitor resistance [48, 49]. Our findings further confirm the potential of targeting mitochondrial metabolism in MM therapy.

Due to the heterogeneity of plasma cells, we divided them into 6 subpopulations and observed a significant expansion in plasma cell_2 and plasma cell_3. We conducted independent modular analyses of plasma cell CNV, differentiation potential, transcription factor, the cell cycle, the expression of high-frequency mutant genes, glucose metabolism, and other factors to examine the notable correlation between plasma cell_2 and the malignant proliferation of MM plasma cells. In addition, we explored new theranostic targets from membrane surface proteomics and kinase omics.

WNK1 has been reported to be a therapeutic target for a variety of tumors [50–52]. We identified WNK1 as a potential therapeutic target for MM. The specific inhibitor WNK-IN-11 can significantly inhibit the growth of MM tumor cells and impede the progression of MM in both subcutaneous and systemic tumor models. And that reduced the burden on tumor-bearing mice and prolonged their survival. In addition, WNK1 was reported to be altered during brucein D-induced apoptosis of MM cells [53]. However, we found that the use of WNK-IN-11 did not cause apoptosis of MM cells.

In conclusion, we performed a scRNA-seq of the B lymphocyte lineage of MM patients and analyzed the heterogeneity of MM plasma cells from various perspectives. Our findings offer insights for future exploration of MM, reveal new theranostic targets, and present innovative ideas for the diagnosis and treatment of MM.

## Supplementary information

supplemental material

Supplemental tables

## Data Availability

Data generated during this study have been deposited in the National Genomic Data Center [54] with the accession numbers HRA004197. All datasets that support the findings of the current study are available from the corresponding author upon reasonable request.

## Code availability

Codes could be accessed, along with the supporting data, upon reasonable request from Y.P.
