# Supplementary material for: Single-cell sequencing analysis of multiple myeloma heterogeneity and identification of new theranostic targets
Source: Cell Death Dis. 2024 Sep 14;15(9):672. doi: 10.1038/s41419-024-07027-4 (PMC11399131; doi:10.1038/s41419-024-07027-4)
Supplement: Supplementary file 1 — supplemental material [file 41419_2024_7027_MOESM1_ESM.pdf]

Supplemental Figures and legends

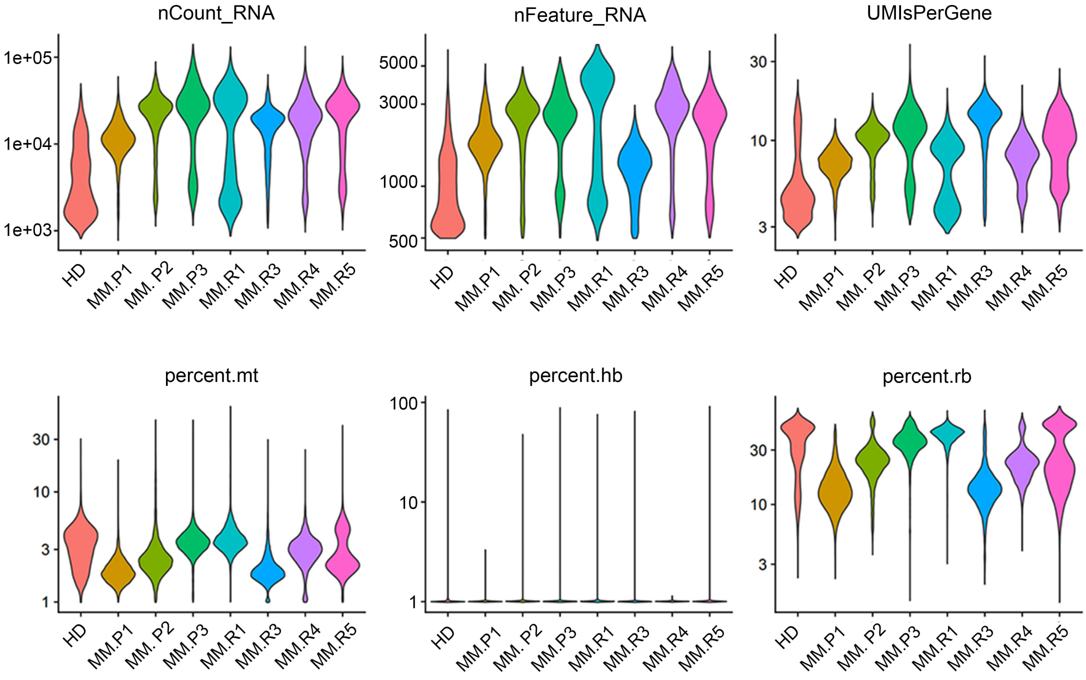


Figure S1

Figure S1 Quality control results of single-cell data.

Ncount_rna is the total number of mRNA molecules. Nfeature_rna is the number of genes. Umispergene is the median of UMI data detected in each cell. Percent.mt is the proportion of genes in mitochondria. Percent.hb is the proportion of erythrocyte gene expression. Percent.rb is the proportion of ribosomal gene expression.

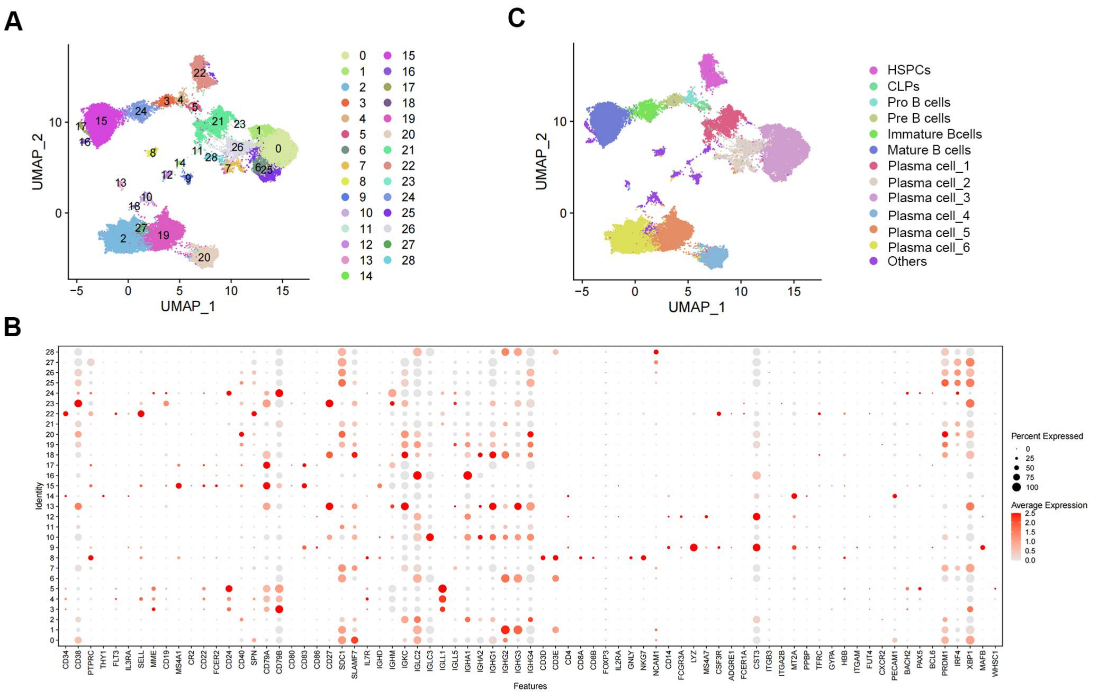


Figure S2

Figure S2 Cell clusters and annotations.

(**A-B**) UMAP representation of single-cell gene expression showing 29 identified cell clusters and 10 identified retinal cell types. Single cells were color-coded by cluster annotation. (**C**) Dot plot showing expression of each celltype characteristic genes.

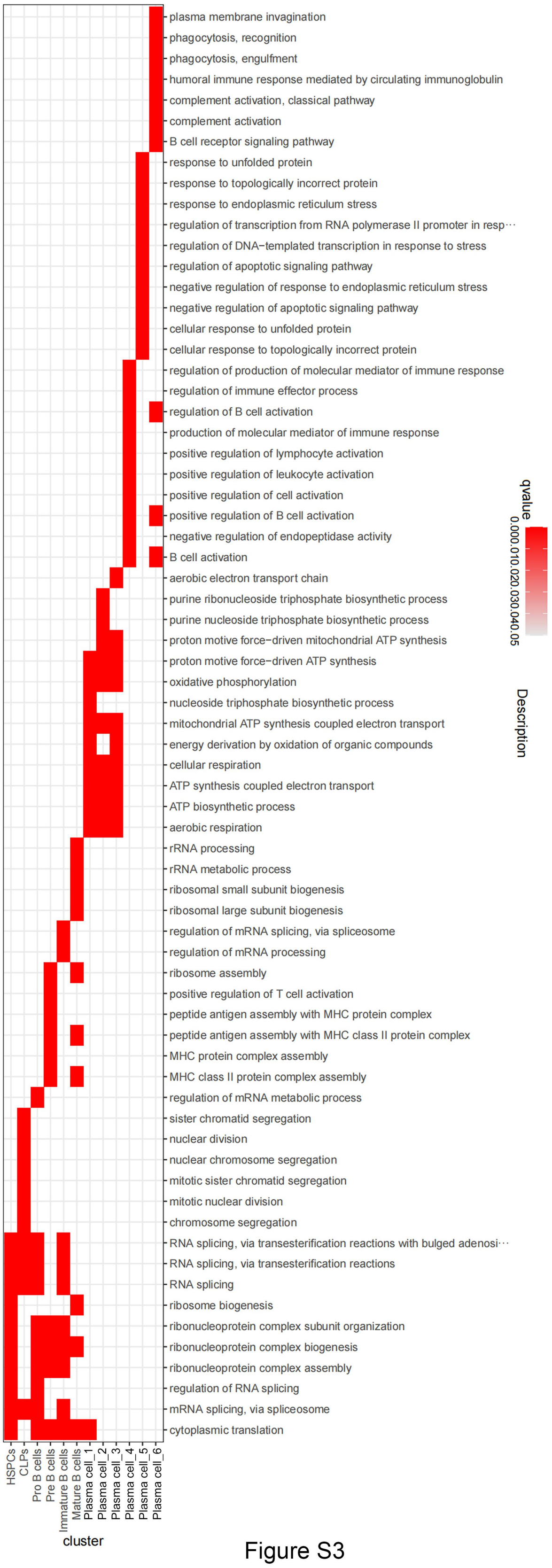


Figure S3

Figure S3 GO enrichment of cell clusters.

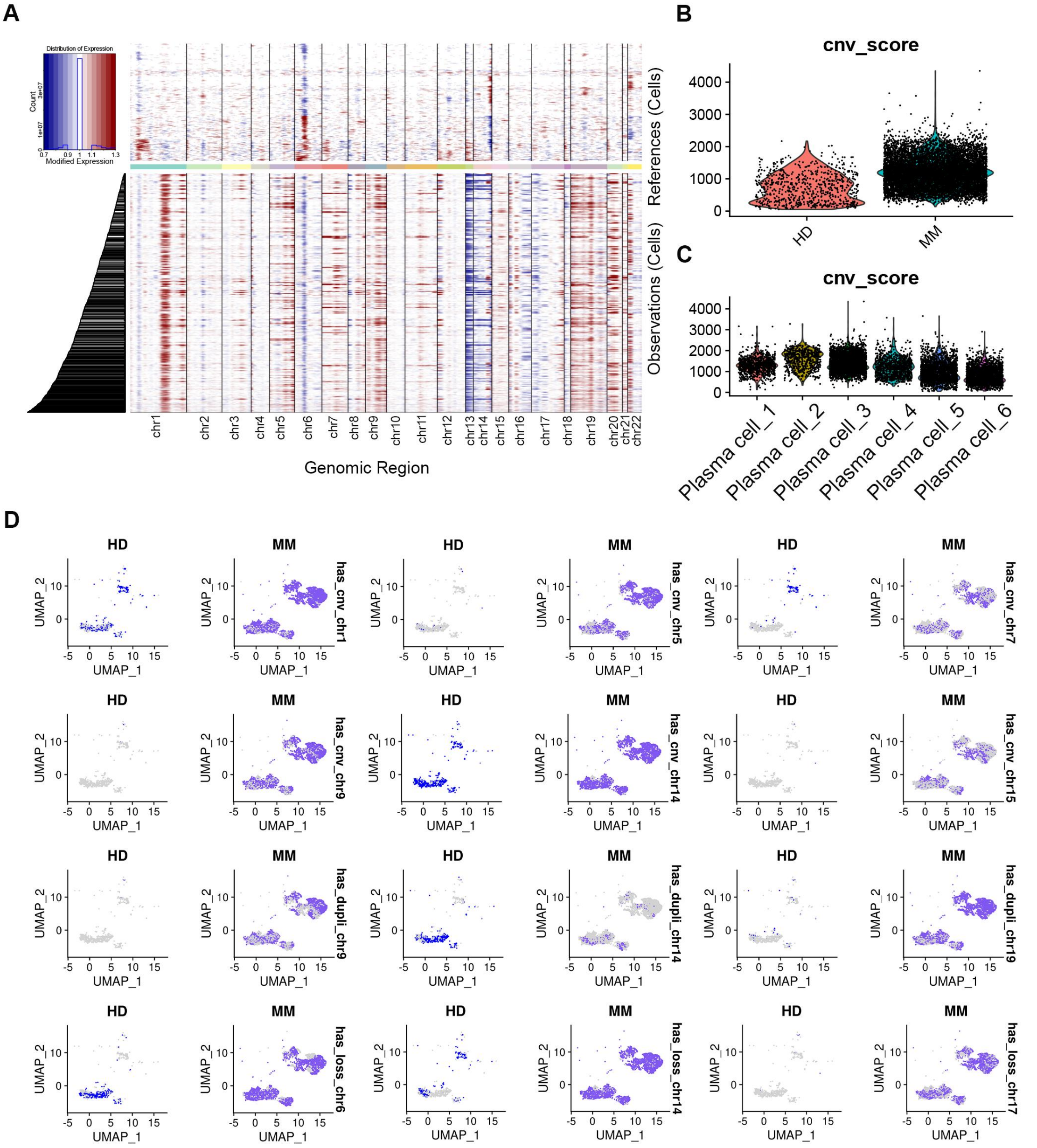


Figure S4

Figure S4 Plasma cells CNV analysis.

(**A**) CNV observation of a genomic region. (**B**) CNV score of HD and MM. (**C**) CNV score of six plasma cell subsets. (**D**) UMAP showing CNV changes between HD and MM.`

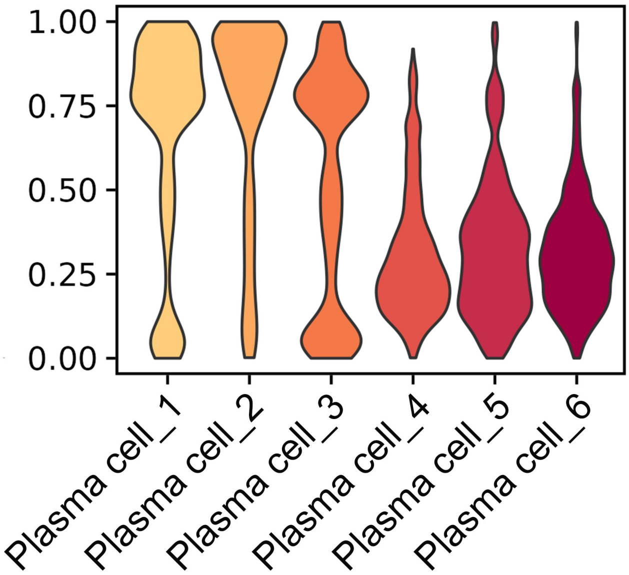


Figure S5

Figure S5 Differentiation potential of plasma cell subpopulations.

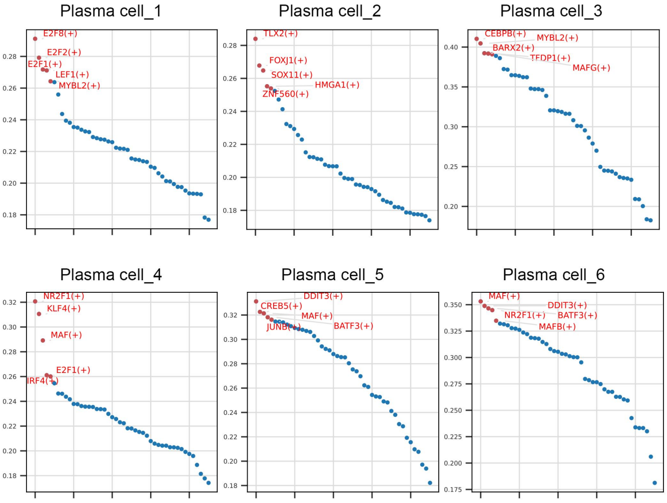


Figure S6

Figure S6 Transcription factors expression of plasma cell subpopulations.

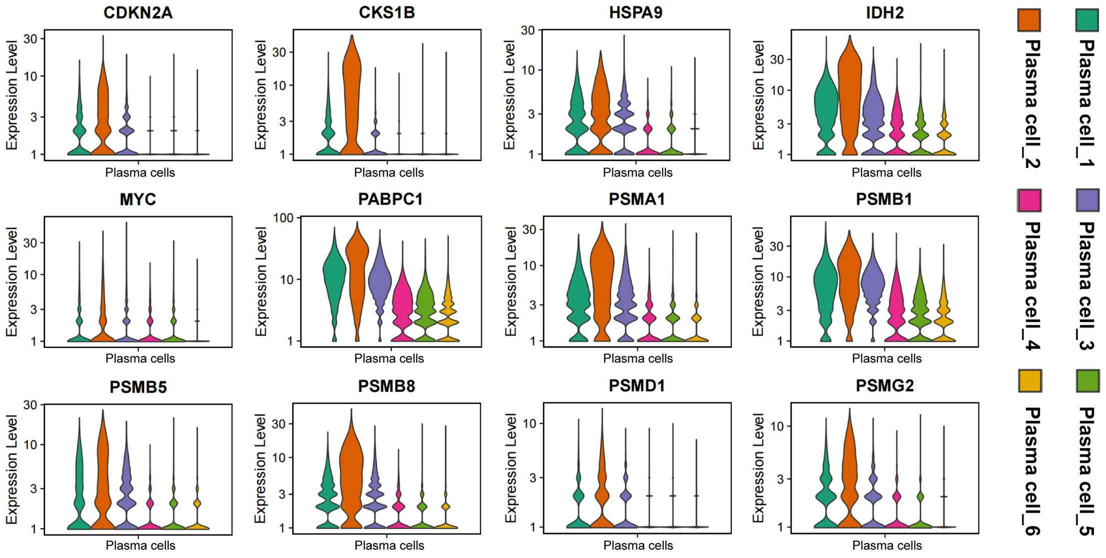


Figure S7

Figure S7 Expression of multiple high-frequency mutated genes in various subpopulations of plasma cells.

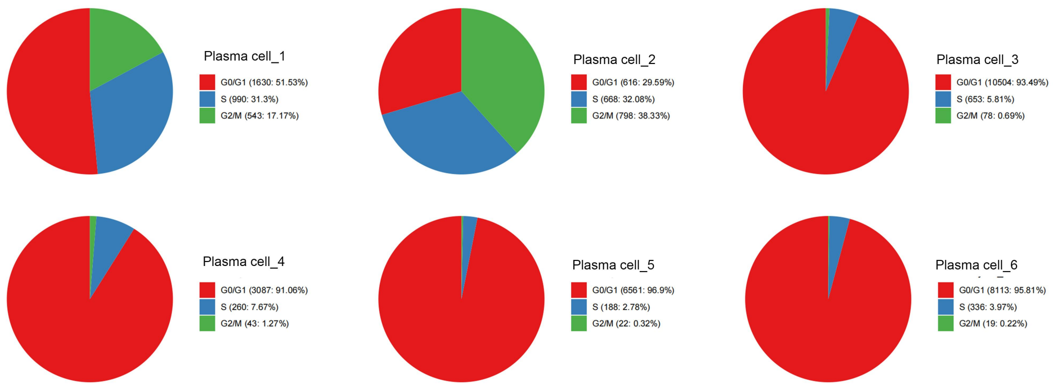


Figure S8

Figure S8 Seurat analysis of the cell cycle distribution in each cell subpopulation.

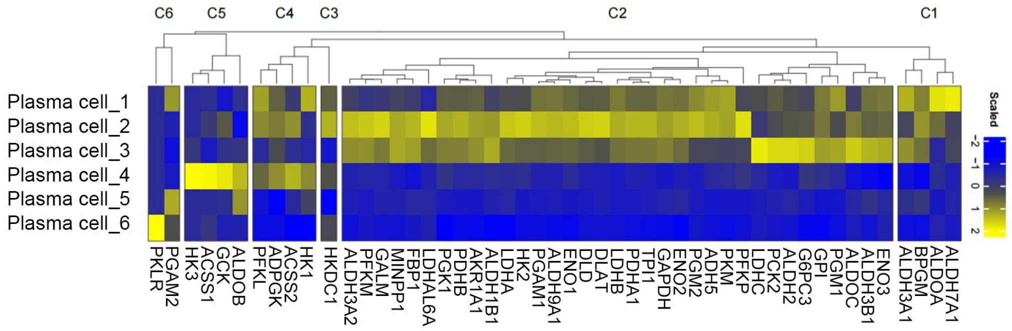


Fugure S9

Figure S9 Glucose metabolism-related gene expression in various subpopulations of plasma cells.

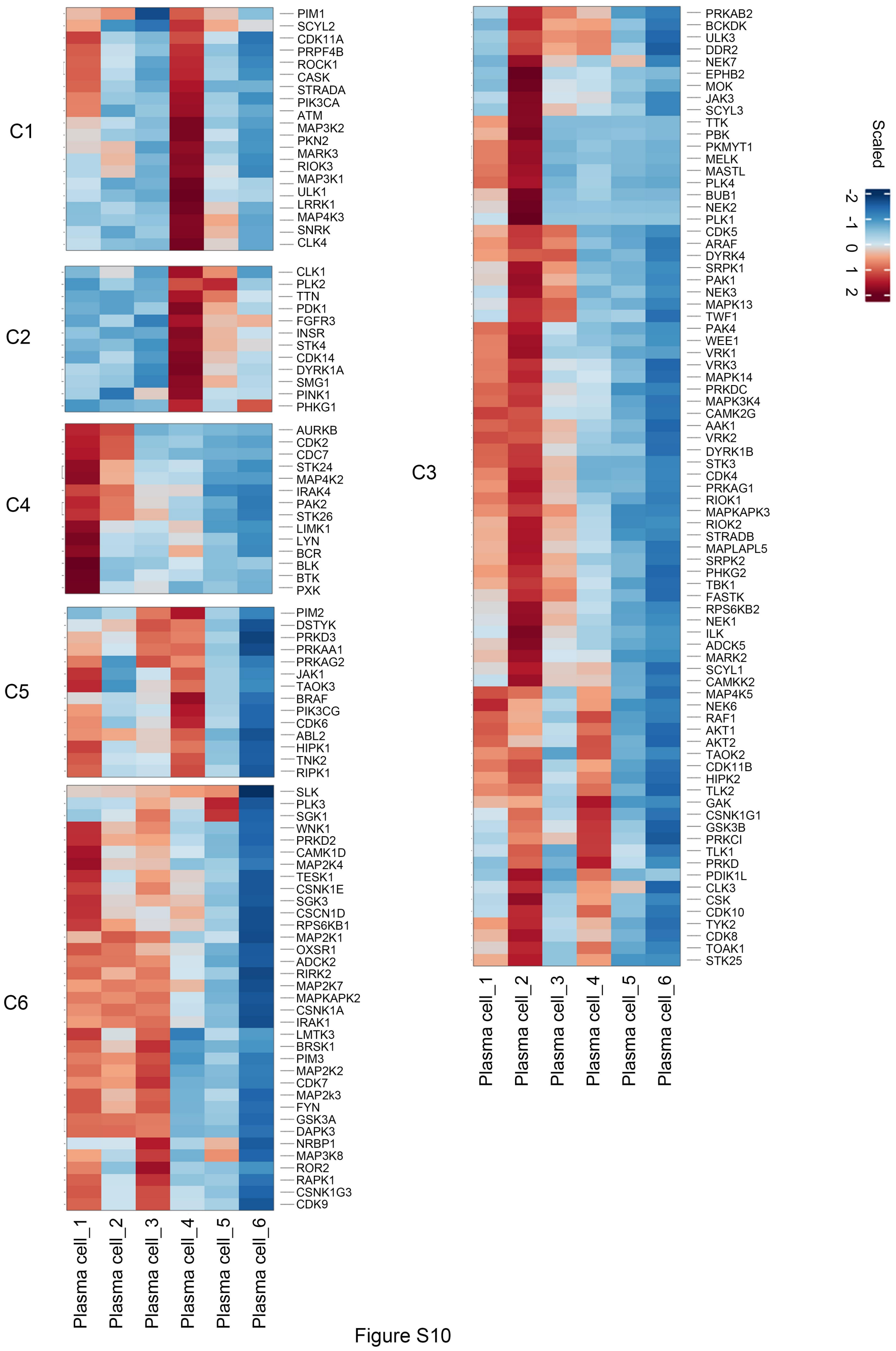


Figure S10

Figure S10 Characteristic expressed kinases in various subpopulations of plasma cells.

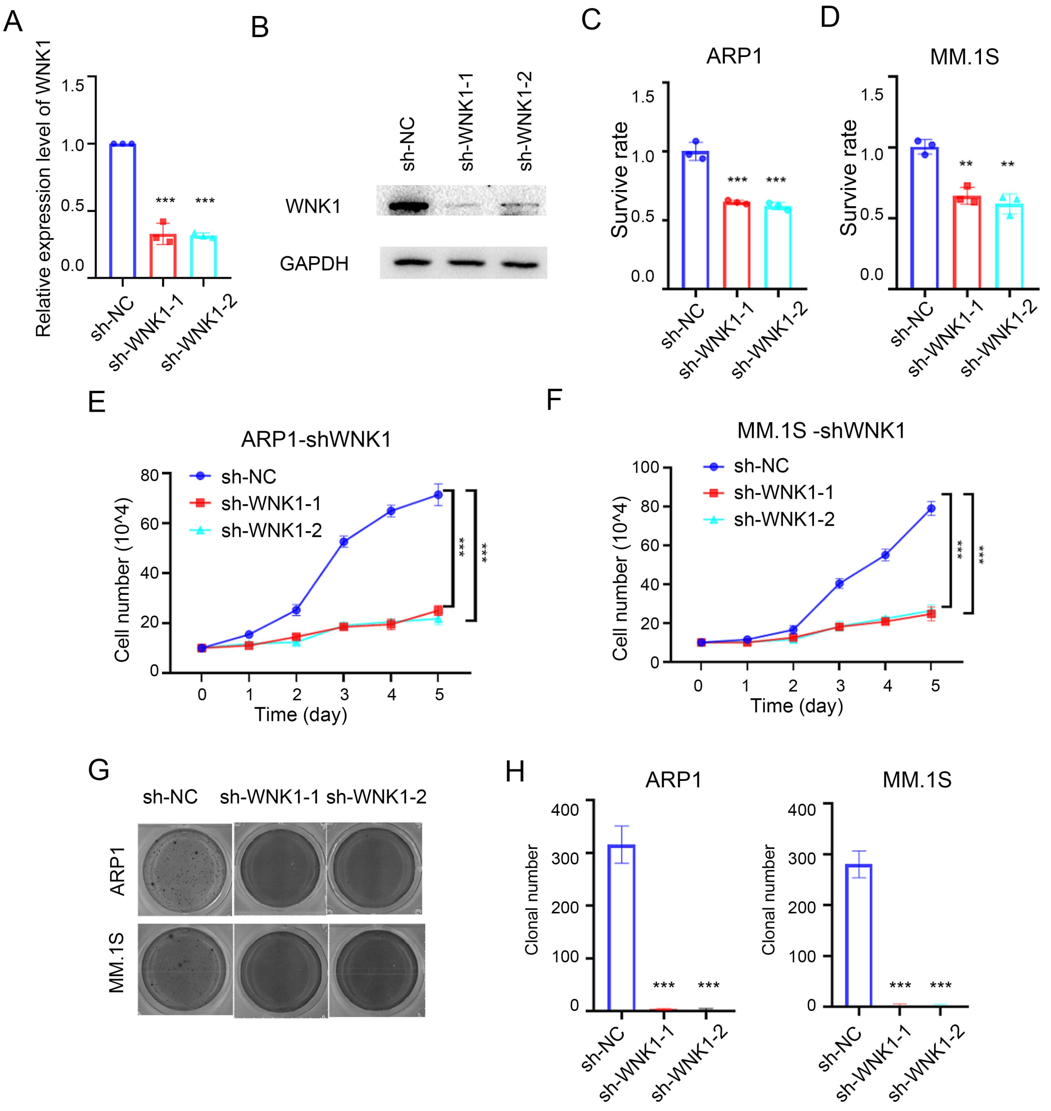


Figure S11

Figure S11 Knockdown of WNK1 significantly inhibited MM cell viability and proliferation.

(A-B) The shRNA virus targeting WNK1 was used to infect ARP1 and MM.1S cells, and cell activity was detected by CCK8. (C) ARP1 and MM.1s cells were infected with WNK1 shRNA virus, and cell proliferation was detected by manual counting. (D) ARP1 and MM.1s cells were infected with WNK1 shRNA virus, and the clonogenic ability of cells was detected by soft agar plate clonogenic assay. Statistical analyses of n=3 independent experiments were assessed. Results are shown as mean ± SD.

*$p$<0.05, **$p$<0.01, ***$p$<0.001.

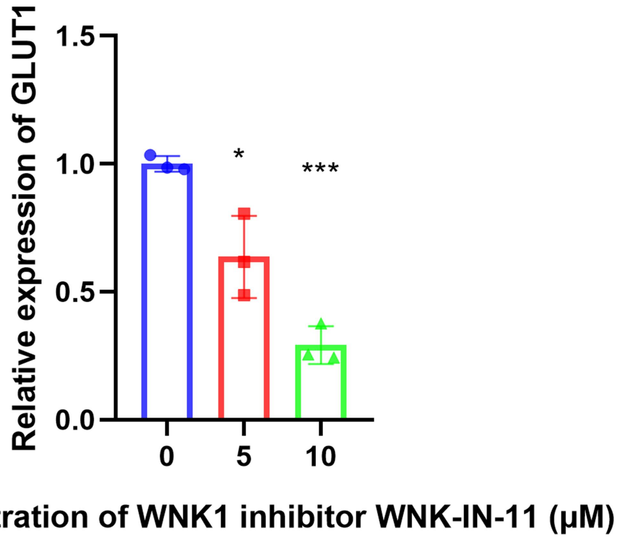


**Figure S12**

Figure S12 Gray quantitative analysis of Figure 5H

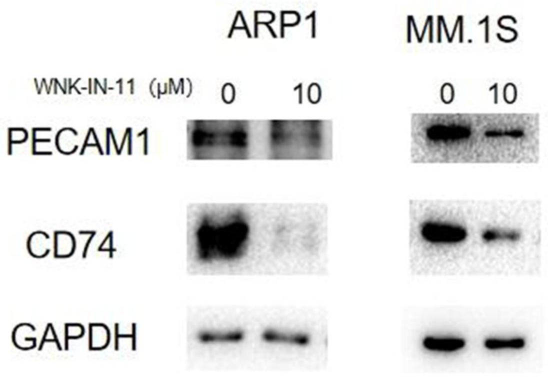


Figure S13

Figure S13 WNK-IN-11 inhibits the expression of signaling molecules PECAM1 and

CD74

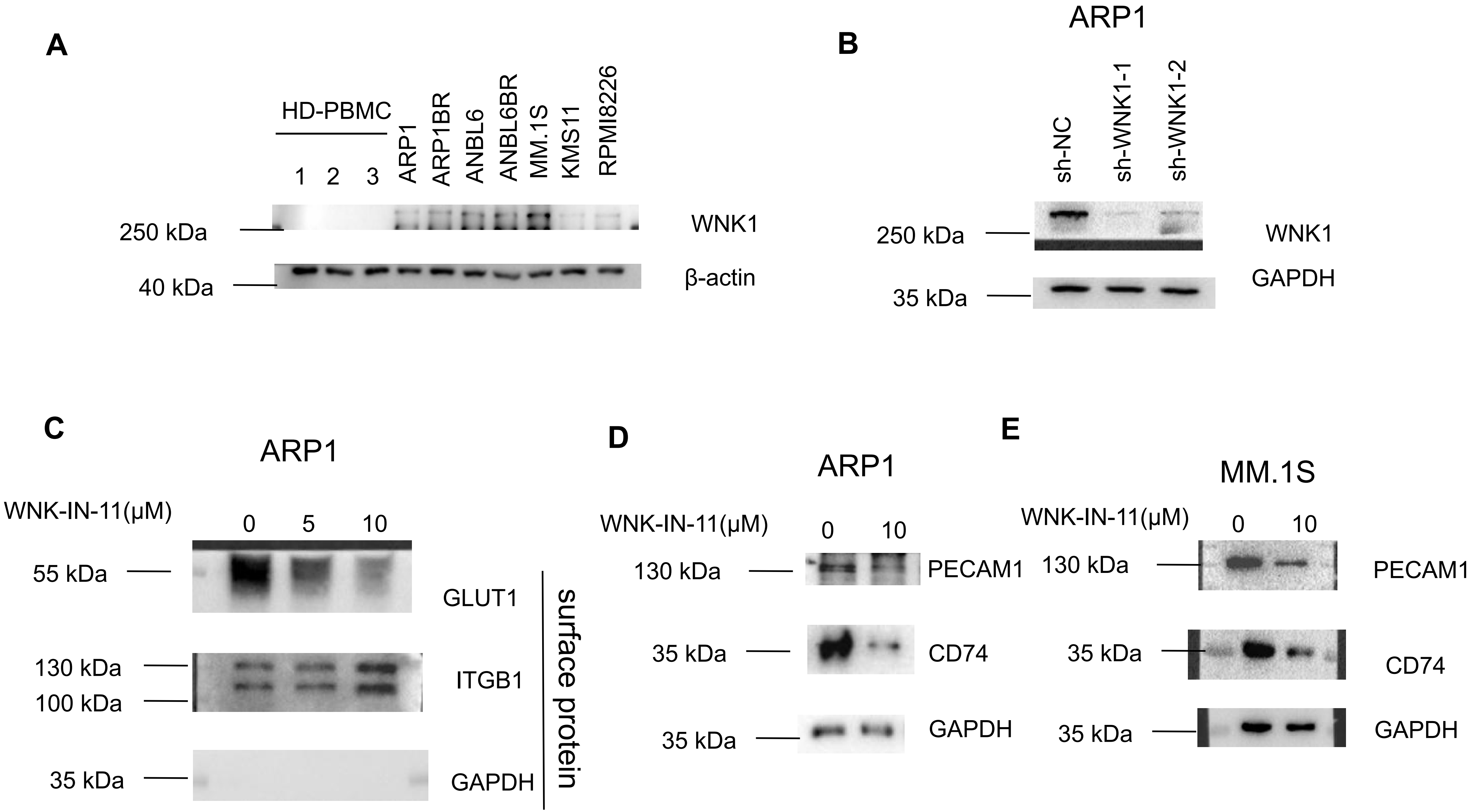

A
HD-PBMC
1
2
3
ARP1
ARP1BR
ANBL6
ANBL6BR
MM.1S
KMS11
RPMI8226
250 kDa
WNK1
40 kDa
β-actin
B
ARP1
sh-NC
sh-WNK1-1
sh-WNK1-2
250 kDa
WNK1
35 kDa
GAPDH
C
ARP1
WNK-IN-11(μM)
0
5
10
55 kDa
GLUT1
130 kDa
100 kDa
ITGB1
35 kDa
GAPDH
surface protein
D
ARP1
WNK-IN-11(μM)
0
10
130 kDa
PECAM1
35 kDa
CD74
35 kDa
GAPDH
E
MM.1S
WNK-IN-11(μM)
0
10
130 kDa
PECAM1
35 kDa
CD74
35 kDa
GAPDH
